# Nucleated red blood cells as predictors of mortality in patients with acute respiratory distress syndrome (ARDS): an observational study

**DOI:** 10.1186/s13613-018-0387-5

**Published:** 2018-03-27

**Authors:** Mario Menk, Lena Giebelhäuser, Gerald Vorderwülbecke, Martina Gassner, Jan A. Graw, Björn Weiss, Mathias Zimmermann, Klaus-D. Wernecke, Steffen Weber-Carstens

**Affiliations:** 1 Department of Anaesthesiology and Operative Intensive Care Medicine (CCM/CVK), Charité – University Medicine Berlin, Corporate Member of Freie Universität Berlin, Humboldt-Universität zu Berlin, and Berlin Institute of Health, Campus Virchow-Klinikum, Augustenburger Platz 1, 13353 Berlin, Germany; 2 Central Institute of Laboratory Medicine, DRK Klinikum Berlin Westend, Spandauer Damm 130, 14050 Berlin, Germany; 3 Charité - University Medicine Berlin, Charitéplatz 1, 10117 Berlin, Germany; 4 Sostana GmbH, Wildensteiner Straße 27, 10318 Berlin, Germany

**Keywords:** ARDS, Nucleated red blood cells, Risk stratification, Predictive value, Outcome

## Abstract

**Background:**

Nucleated red blood cells (NRBCs) in critically ill patients are associated with increased mortality and poor outcome. The aim of the present study was to evaluate the predictive value of NRBCs in patients with acute respiratory distress syndrome (ARDS).

**Methods:**

This observational study was conducted at an ARDS referral center and included patients from 2007 to 2014. Daily NRBC counts were assessed and the predictive validity of NRBCs on mortality was statistically evaluated. A cutoff for prediction of mortality based on NRBCs was evaluated using ROC analysis and specified according to Youden’s method. Multivariate nonparametric analysis for longitudinal data was applied to prove for differences between groups over the whole time course. Independent predictors of mortality were identified with multiple logistic and Cox’ regression analyses. Kaplan–Meier estimations visualized the survival; the corresponding curves were tested for differences with the log-rank test.

**Results:**

A total of 404 critically ill ARDS patients were analyzed. NRBCs were found in 75.5% of the patients, which was associated with longer length of ICU stay [22 (11; 39) vs. 14 (7; 26) days; *p *< 0.05] and higher mortality rates (50.8 vs. 27.3%; *p *< 0.001). Logistic regression analysis with mortality as response showed NRBC positivity per se to be an independent risk factor for mortality in ARDS with a doubled risk for ICU death (OR 2.03; 95% CI 1.16–3.55; *p *< 0.05). Also, NRBC value at ICU admission was found to be an independent risk factor for mortality (OR 3.25; 95% CI 1.09–9.73, *p* = 0.035). A cutoff level of 220 NRBC/µl was associated with a more than tripled risk of ICU death (OR 3.2; 95% CI 1.93–5.35; *p *< 0.0001). ARDS patients below this threshold level had a significant survival advantage (median survival 85 days vs. 29 days; log rank *p *< 0.001). Presence of a severe ARDS was identified as independent risk factor for the occurrence of NRBCs > 220/µl (OR 1.81; 95% CI 1.1–2.97; *p *< 0.05).

**Conclusions:**

NRBCs may predict mortality in ARDS with high prognostic power. The presence of NRBCs in the blood might be regarded as a marker of disease severity indicating a higher risk of ICU death.

## Background

Prediction of outcome in critically ill patients with the acute respiratory distress syndrome (ARDS) is of major importance for appropriate treatment decisions and resource allocation. Several variables, such as the oxygenation index (OI) or the ratio of arterial partial pressure of oxygen divided by the fraction of inspired oxygen (PaO_2_/FiO_2_ ratio), have been screened for their predictive value in ARDS patients [1, 2]. Also, several definitions of the syndrome have been used by clinicians not only to categorize ARDS patients, but also in an attempt to predict mortality [3, 4]. However, prognostication tools in ARDS are limited and none of the existing parameters, variables or proposed definitions fully resolves the problem.

Nucleated red blood cells (NRBCs) are progenitor cells of the mammalian erythropoietic lineage. These cells are not usually present in the peripheral blood of healthy adults, but might occur in the blood stream in certain disease states and in critical illness [5–7]. Increased erythropoietin and high levels of pro-inflammatory cytokines (such as IL-6) are associated with the occurrence of NRBCs in the blood [8]. Further, arterial hypoxemia might be a major triggering factor for NRBCs [9]. Although the exact mechanism by which NRBCs are finally released from the bone marrow into the circulation remains unclear, their presence in the peripheral blood is a valid marker of disease severity and increased mortality [5, 10–13]. For example, in sepsis, septic shock and in cardiac patients, NRBC positivity is suggested as a marker predicting all-cause in-hospital mortality [5, 14].

The predictive value of NRBCs in patients with ARDS has not yet been evaluated. ARDS might be of special interest in this respect, as the syndrome usually combines the main triggering factors for releasing NRBCs, i.e., arterial hypoxemia and severe systemic inflammation. Therefore, the present study tested the hypothesis that the presence of NRBCs sufficiently predicts mortality in severely affected ARDS patients.

## Methods

### Setting and patients

This retrospective, observational analysis was conducted at a 14-bed intensive care unit (ICU) of the department of Anesthesiology and Intensive Care Medicine, Charité University Medicine Berlin, a national referral center for treatment of ARDS and part of the German ARDS network (http://www.ardsnetwork.de). After written consent from the hospital ethics commission (EA2/172/17), all adult patients admitted for ARDS according to the Berlin definition [4] between January 2007 and December 2014 entered the study. Excluded were patients aged < 18 years, those that were re-admitted after hospital discharge and patients who died within the first 24 h after ICU admission. Also excluded were patients for whom NRBC values were not available for any reason. ARDS treatment followed our local standard operating procedures describing indications and duration of advanced therapeutic interventions including proning, inhalation of nitric oxide (NO) and the implementation of extracorporeal gas exchange devices such as extracorporeal membrane oxygenation (ECMO) or pumpless extracorporeal lung assist (pECLA), as published previously [15].

### Data collection

Clinical routine data were extracted from the two electronic patient data management systems in use at the hospital (COPRA, Sasbachwalden, Germany; and SAP, Walldorf, Germany). In addition to basic demographic and anamnestic data (sex, age, height, weight), we assessed ICU admission scores (APACHE II, SAPS II, SOFA and TISS-28 scores) and severity of ARDS according to the Berlin definition (mild, moderate, severe) [4]. Being major clinical causes leading to ARDS, we differentiated between pneumonia, sepsis of extra-pulmonary origin, trauma, immunodeficiency and “acute-on-chronic” (i.e., patients with a preexisting chronic pulmonary disease with an acute exacerbation), as described previously [16]. Also assessed on ICU admission were parameters of pulmonary gas exchange and mechanical ventilation such as the peak inspiratory pressure (Ppeak), mean airway pressure (Pmean), positive end-expiratory pressure (PEEP), the inspiratory fraction of oxygen (FiO_2_), arterial partial pressure of oxygen (PaO_2_) and pulmonary compliance. The utilization and respective duration of extracorporeal lung assist devices (ECMO or pECLA), the duration of mechanical ventilation, ICU length of stay and all-cause ICU mortality were recorded in order to characterize the patient population.

### Laboratory tests

Anticoagulated EDTA-blood samples were obtained from patients at least once daily until discharge from the ICU. NRBC count was routinely measured in the white cell nucleated (WNR) channel of a Sysmex XN-9000 hematology analyzer (Sysmex, Kobe, Japan) using fluorescence flow cytometry as method of determination. Counts are reported in NRBC cells (*n*)/µl. In case of more than one NRBC measurement per day, the highest value was taken for analysis. We also used the highest NRBC value during the ICU stay for each patient for analyses over the course of time, receiver operator curves (ROC), linear regression, Cox’ regression and survival analysis. For binary analysis and initial grouping of patients, NRBC positivity was defined as any NRBC value above zero at any time during the ICU stay.

### Statistical analysis

Discrete variables are given as absolute numbers, counts or percentage, and continuous variables as medians with 25; 75 percentiles, as indicated in brackets. For demographics and patient characteristics, statistical differences between the groups were assessed using Fisher’s exact test for categorical variables or the Mann–Whitney test for continuous variables, when appropriate. Predictive validity of NRBC measurements for mortality was assessed using receiver operator curves (ROC) and corresponding results for the area under the curve (AUC). From this, a cutoff for mortality based on NRBC was specified according to Youden’s method (representing the highest sum of sensitivity and specificity) [17]. This cutoff value was used to allocate patients to one of two groups (i.e., above or below the calculated cutoff value) for subsequent analyses of predictivity. Multiple logistic regression analysis with stepwise backward selection was used to test multivariately for factors that influence mortality. Kaplan–Meier estimations were used to visualize differences in survival between patients using the calculated cutoff value. Differences in survival between patients under or above the calculated cutoff value were tested using the log-rank test. In order to test multivariately for factors that influence survival, Cox regression was applied with stepwise backward selection, including variables that showed a statistically significant impact in the univariate analyses. Multivariate, nonparametric analysis for longitudinal data in a two-factorial design (first factor (independent): group, second factor (dependent): time) was applied for analysis of data over the whole time course. Those results are given as relative effects, as well as corresponding *p*-values, as indicated. The relative effect presents (in a scale between 0 and 1, according to a probability) the specific treatment effect of the regarded group, relative to all groups, therefore to a “mean” treatment effect. As our study was retrospective, we did not calculate the sample size in advance. Nevertheless, a post hoc power calculation for multiple regression analysis with different effect sizes from our calculations with the logistic regression (0.04 acc. to Cox and Snell and 0.05 acc. to Nagelkerke), 4 predictors, *α* = 5% and power = 80%, resulted in 304 and 244 patients, respectively (calculations with G*Power, Version 3.1.7, Copyright © 1992–2013). Statistical analyses were performed with IBM SPSS Statistics, Version 24 (SPSS, Chicago, IL, USA) and The R Project for Statistical Computing, Version 3.4.0 (2017-04-21), Copyright © 2017 The R Foundation for Statistical Computing and GraphPad PRISM version 7 (San Diego, CA, USA). A two-tailed *p* value < 0.05 was considered statistically significant. All tests should be understood as constituting exploratory analysis, such that no adjustments for multiple testing have been made.

## Results

### Patient characteristics

A total of 458 critically ill patients admitted for ARDS were treated between January 2007 and December 2013 and were screened for this study. Of these, 54 patients were excluded due to missing data on NRBC values, yielding a final study population of 404 patients. Of these, 305 patients (75.5%) were considered NRBC positive (i.e., any NRBC value above zero at any time during the ICU stay). Characteristics of the study population at baseline (grouped by NRBC positivity yes/no) are presented in Table 1. NRBC positivity was associated with significantly higher severity scores at ICU admission. Also, NRBC-positive patients were more likely to suffer from more severe forms of ARDS and showed a significantly lower pulmonary compliance, a longer duration of mechanical ventilation and a prolonged ICU stay. No significant differences were found between NRBC-positive and NRBC-negative patients regarding the parameters of mechanical ventilation and pulmonary gas exchange. Also, the etiology of ARDS showed no significant difference between NRBC-positive and NRBC-negative patients. Over time, extracorporeal gas exchange was more often implemented in NRBC-positive patients, with veno-venous ECMO being the predominant procedure. While the overall ICU mortality was 45%, NRBC positivity was associated with a significantly higher mortality rate compared with NRBC-negative patients (50.8 vs. 27.3%; *p *< 0.001).

**Table 1:** Patient characteristics and comparison between NRBC-positive and NRBC-negative ARDS patients

	All (*n* = 404)	NRBC positive (*n* = 305; 75.5%)	NRBC negative (*n* = 99; 24.5%)	*p* value
Basic characteristics				
Age (years)	50 (37; 61)	49 (38; 61.5)	49 (33; 60)	n.s.
Sex (male) (*n*)	265 (65.6%)	191 (62.6%)	74 (74.7%)	< 0.05*
Body mass index (kg/m^2^)	26.2 (23.3; 30.8)	26.2 (23.3; 30)	26.5 (23.4; 31.9)	n.s.
Severity of illness scores at ICU admission				
SAPS II	55 (39; 70)	56 (40; 71)	53 (36.5; 66)	n.s.
APACHE II	27 (20; 35)	29 (21; 36)	25.5 (19; 33)	< 0.05*
SOFA II	12 (9; 15)	12 (9; 15)	11 (8; 13)	< 0.001***
TISS-28	51 (43; 58)	51 (43; 58)	50 (43; 56)	n.s.
Severity of ARDS				
Mild (*n*)	12 (3%)	7 (2.3%)	5 (5.1%)	n.s.
Moderate (*n*)	174 (43.1%)	123 (40.3%)	51 (51.5%)	< 0.05*
Severe (*n*)	207 (51.2%)	168 (55.1%)	39 (39.4%)	< 0.05*
Pulmonary gas exchange and mechanical ventilation (at ICU admission)				
Ppeak (cm H_2_O)	35 (32; 39)	35 (32; 39)	34 (31; 38)	n.s.
Pmean (cm H_2_O)	24 (21; 28)	24 (21.5; 28)	23.4 (21; 27)	n.s.
PEEP (cm H_2_O)	17 (15; 20)	17 (15; 20)	16.7 (15; 20)	n.s.
Delta *P* (cm H_2_O)	18 (14.7; 21.6)	18 (14.7; 21.2)	18.7 (15; 22)	n.s.
Tidal volume/PBW (ml/kg)	5.9 (4.7; 7.4)	5.8 (4.3; 7.2)	6.75 (5.2; 7.9)	n.s.
FiO_2_	93 (70; 100)	93 (70; 100)	91.5 (70; 100)	n.s.
PaO_2_ (mmHg)	135 (111; 170)	136 (113; 179)	123 (108; 165)	n.s.
PaCO2 (mmHg)	52 (42; 64)	52 (43; 63.2)	53.3 (41.8; 67.7)	n.s.
PaO_2_/FiO_2_	163 (126; 204)	165 (135; 225)	139 (116; 183)	n.s.
OI	17.5 (11.8; 29.1)	17.6 (12; 28.9)	17.1 (11; 29.4)	n.s.
Pulmonary compliance (ml/cmH_2_O)	28.4 (19.4; 40.7)	26.55 (17.5; 38.3)	34.1 (24.7; 44.1)	< 0.001***
Mechanical ventilation (hours)	448 (193; 743)	484 (242; 825)	308 (154; 527.25)	< 0.001***
ICU length of stay (days)	20 (10; 35)	22 (11; 38.5)	14 (7; 26)	< 0.05*
Etiology of ARDS				
Pneumonia (*n*; %)	221 (54.7%)	168 (55.1%)	53 (53.5%)	n.s.
Sepsis (*n*; %)	25 (6.2%)	20 (6.6%)	5 (5.1%)	n.s.
Immune deficiency (*n*; %)	61 (15.1%)	47 (15.4%)	14 (14.1%)	n.s.
Acute-on-chronic (*n*; %)	46 (11.4%)	35 (11.5%)	11 (11.1%)	n.s.
Trauma (*n*; %)	26 (6.4%)	18 (5.9%)	8 (8.1%)	n.s.
Others (*n*; %)	14 (3.5%)	11 (3.6%)	3 (3%)	n.s.
Extracorporeal lung support				
ECLS (*n*; %)	231 (57.2%)	192 (63%)	39 (39.4%)	< 0.001***
pECLA (*n*; %)	107 (26.5%)	79 (25.9%)	28 (28.3%)	n.s.
ECMO (*n*; %)	156 (38.6%)	143 (46.9%)	13 (13.1%)	< 0.001***
ECMO and pECLA (*n*; %)	32 (7.9%)	30 (9.8%)	2 (2%)	< 0.001***
ICU mortality	182 (45%)	155 (50.8%)	27 (27.3%)	< 0.001***

Discrete variables are presented as absolute numbers, median or percentage and were analyzed with Fisher’s exact test for independent groups. Continuous variables are presented as median and (25; 75) percentiles and were analyzed with the Mann–Whitney test for independent groups. NRBC positivity was defined as any NRBC value above zero at any time during the ICU stay

*NRBC* nucleated red blood cells; *SAPS II* Simplified Acute Physiology Score II; *APACHE* II Acute Physiology And Chronic Health Evaluation II; *SOFA* Sequential Organ Failure Assessment, *TISS* Therapeutic Intervention Scoring System. Severity of ARDS according to the “*Berlin*-*definition of ARDS*”; *Ppeak* peak inspiratory pressure, *Pmean* mean airway pressure; *PEEP* positive end-expiratory pressure, delta *P* driving pressure; *PBW* predicted body weight; *FiO*_*2*_ fraction of inspired oxygen, *PaO*_*2*_ arterial partial pressure of oxygen, *PaCO*_*2*_ arterial pressure of carbon dioxide; *OI* oxygenation index FiO_2_/PaO_2_* Pmean; *ICU* intensive care unit; *ECLS* extracorporeal lung support; *pECLA* pumpless extracorporeal lung assist, *ECMO* extracorporeal membrane oxygenation

*p < 0.05; ***p < 0.001

### NRBC: maximum counts and development over time

**Fig. 1:**
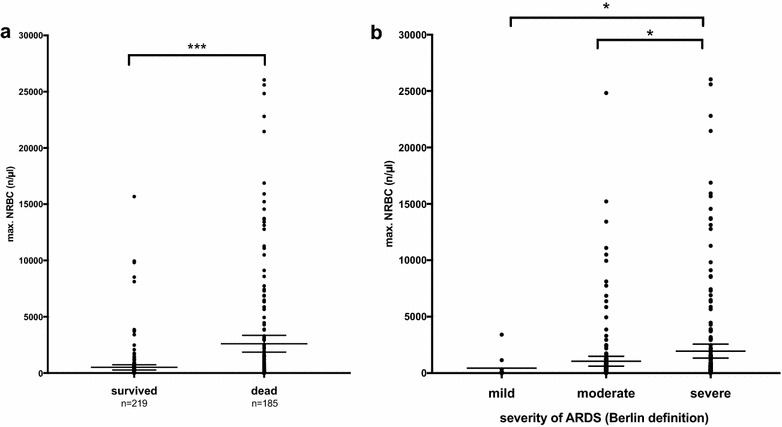
Maximum concentration of nucleated red blood cells (NRBCs) in the blood of ARDS patients grouped by **a** survivors and non-survivors and **b** severity of lung failure according to the Berlin definition of ARDS (mild, moderate, severe); *n* = 404. Mann–Whitney test, ***p < 0.001; *p < 0.05

**Fig. 2:**
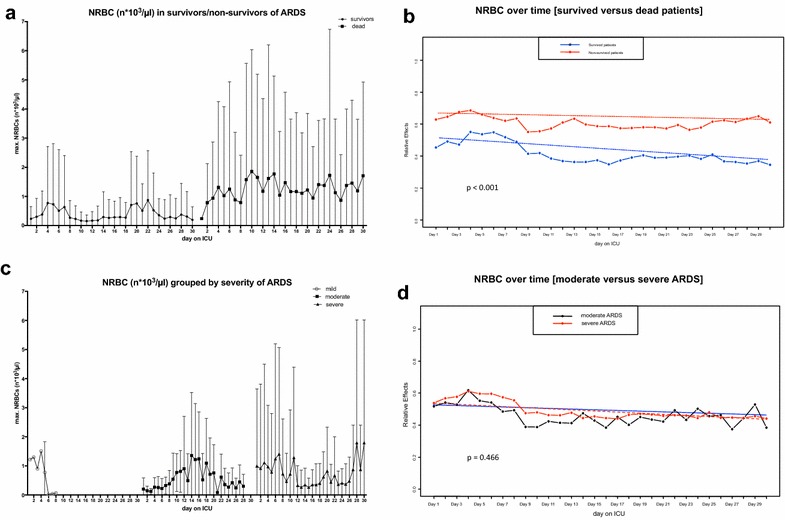
**a** Concentration of NRBCs in the blood of ARDS patients on the first consecutive 30 days on the ICU grouped by survivors and non-survivors. Data are presented as mean ± standard deviation (SD); *n* = 404. **b** Development of NRBC counts over the course of time in survivors (blue solid line; blue dashed line indicates trend) and non-survivors of ARDS (red solid line; red dashed line indicates trend) depicted as relative effect over time. The relative effect presents (in a scale between 0 and 1, and according to a probability) the specific treatment effect of the regarded group, relative to all groups, therefore to a “mean” treatment effect. Multivariate nonparametric analysis for longitudinal data, *p* < 0.001 for comparison between the two indicated groups. **c** Concentration of NRBCs in the blood of ARDS patients on the first consecutive 30 days on the ICU grouped by severity of lung failure according to the Berlin definition of ARDS (mild, moderate, severe). Data are presented as mean ± standard deviation (SD); *n* = 404. **d** Development of NRBC counts over the course of time in severe ARDS (red solid line; red dashed line indicates trend) and moderate ARDS (black solid line; black dashed line indicates trend) depicted as relative effect over time. The relative effect presents (in a scale between 0 and 1, and according to a probability) the specific treatment effect of the regarded group, relative to all groups, therefore to a “mean” treatment effect. Multivariate nonparametric analysis for longitudinal data, *p* = 0.466 for comparison between the two indicated groups. The number of patients with mild ARDS was too low for statistical analysis over time; therefore mild ARDS has been excluded

Overall, ARDS survivors had significantly lower values of maximum NRBC counts in the peripheral blood during the ICU stay than patients who did not survive (surviving patients: 60 (0; 270)/µl; non-surviving patients: 450 (75; 26,000)/µl; *p *< 0.001); (Fig. 1a). When classified according to the Berlin definition of ARDS, patients with moderate or severe ARDS had significantly higher NRBC counts than patients with mild ARDS (mild ARDS: 45 (0; 280)/µl; moderate ARDS: 110 (0; 592)/µl; severe ARDS: 210 (30; 1140)/µl; *p *< 0.05) (Fig. 1b). When considering the first consecutive 30 days on the ICU, NRBC values of non-surviving patients were consistently higher than in surviving patients (Fig. 2a). Multivariate nonparametric longitudinal analysis revealed a significant difference between the groups (survivors vs. non-survivors; *p *< 0.001) and a significantly different trend between the groups over the time course (*p *< 0.001) (Fig. 2b). This trend effect was largely attributable to a significant decrease in NRBC counts in surviving patients (*p *< 0.001), whereas there was hardly any change of NRBC counts over the course of time in non-surviving patients (*p *= 0.42). Further, NRBC peak values tended to occur earlier in surviving patients, whereas non-surviving patients tended to have a NRBC peak a few days later (survivors: 5 (3, 9) days; non-survivors: 7 (3, 17) days; *p *= 0.08).

When classified according to the Berlin definition of ARDS, NRBCs disappeared rapidly from the peripheral blood in mild ARDS, but were constantly detectable in patients with moderate and severe ARDS (Fig. 2c). Over the time course, NRBC counts decreased significantly in both moderate and severe ARDS (*p *< 0.001), but there were no significant differences between these two groups (moderate vs. severe ARDS; *p *= 0.466) (Fig. 2d).

### NRBCs and mortality

**Fig. 3:**
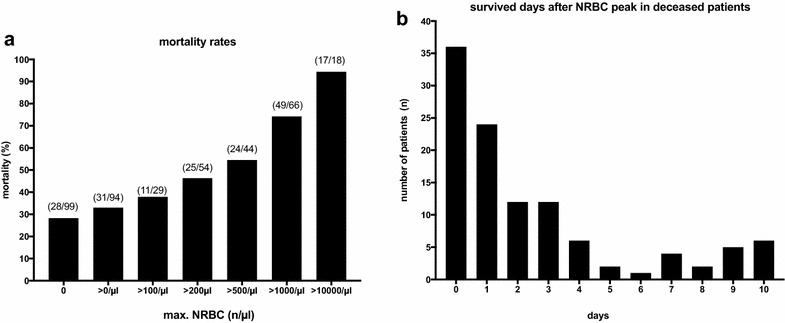
**a** ICU mortality rates of ARDS patients in relation to the maximum concentration of nucleated red blood cells (NRBCs) in the peripheral blood. Numbers in parenthesis denote the ratio of deceased patients to all patients with the respective NRBC concentration. **b** Frequency distribution of the number of survived days after reaching the individual NRBC peak value in deceased patients. Data are presented as a histogram indicating the number of patients in relation to survived days

Increasing levels of NRBCs were associated with higher ICU mortality rates in ARDS patients (Fig. 3a). Overall, the mortality rate was 28% in NRBC-negative patients but increased to almost 100% when NRBC peak values of more than 10.000 per µl were present. In deceased patients, the median survival time was 3 (0; 12) days after reaching the NRBC peak value. However, most of these patients died within the first 24 h after the (individual) NRBC peak (Fig. 3b). Moreover, in deceased patients the NRBC peak value correlated negatively with the remaining survival time. This means that higher NRBC peak values correlated with significantly shorter survival times in non-surviving patients (Spearman’s rho = − 0.31; *p *< 0.001).

Multiple logistic regression analysis including potentially confounding factors for mortality (i.e., age, gender, ARDS severity, implementation of ECMO, APACHE, SOFA, Ppeak, pulmonary compliance) showed NRBC positivity per se to be an independent risk factor for mortality with a more than doubled risk for ICU death (OR 2.03; 95% CI 1.16–3.53; *p* = 0.013). An increase in NRBC value by 1000/µl generated an increase of 19% in the mortality risk. Further, the NRBC value at ICU admission was also found to be an independent predictor of mortality, yielding a more than threefold risk of ICU death (OR 3.25; 95% CI 1.09–9.73; *p* = 0.035). The APACHE II score and pulmonary compliance were also identified as independent risk factors for ICU mortality. In contrast, severity of ARDS, the implementation of extracorporeal gas exchange with ECMO, gender and age did not prove to be independent predictors of mortality (Table 2).

**Table 2:** Multivariate logistic regression and Cox regression analyses of risk factors influencing ICU mortality

Multivariate logistic regression	*p* value	OR	95% CI
APACHE II (admission)	< 0.001	1.04	1.02–1.07
pulmonary compliance (admission)	< 0.001	0.95	0.94–0.97
NRBCs (at ICU admission)	0.035	3.25	1.09–9.73
NRBC cutoff (220/µl)	< 0.001	3.21	1.93–5.35

Cox regression	*p* value	HR	95% CI
APACHE II (admission)	< 0.05	1.02	1.00–1.04
pulmonary compliance (admission)	< 0.001	0.97	0.96–0.99
NRBC > 220/µl	< 0.05	1.41	1.00–1.99

Parameters considered in the multivariate regression models using backwards selection that had significant impact in univariate analyses: age, gender; ICU admission scores (APACHE, SOFA); severe ARDS (yes/no); *P* peak (peak ventilatory pressure); pulmonary compliance; NRBC (yes/no > 220/µl); (ECMO (yes/no). Data of 404 patients were considered. NRBC cutoff 220/µl was used for analysis. *OR* odds ratio; *CI* confidence interval; *HR* hazard ratio; *APACHE II* Acute Physiology And Chronic Health Evaluation II; *NRBC* nucleated red blood cells

Using NRBCs in the regression model, we found an improvement in model discrimination for mortality with respect to correct allocations from 66.4 to 71.1% (4.7% improvement, full model) and from 66.4 to 72.0% (5.6% improvement, selected model), respectively.

### Predictive validity

**Fig. 4:**
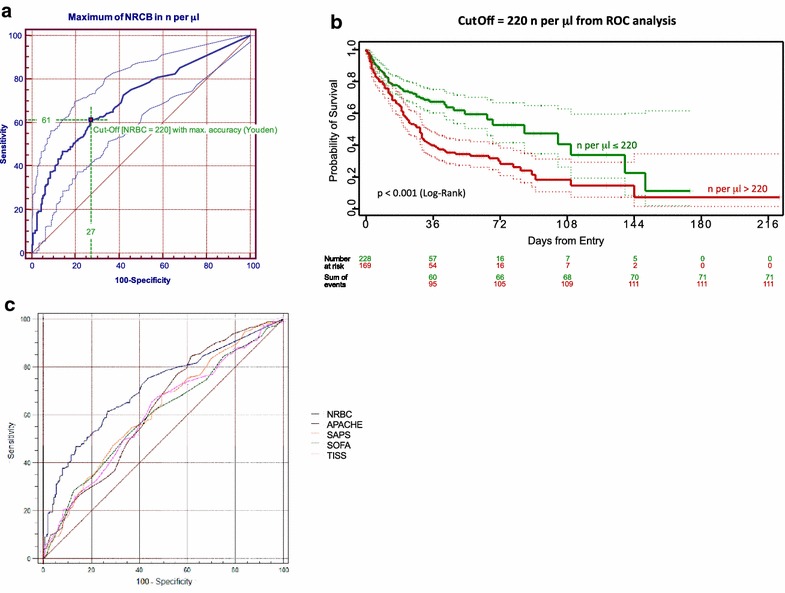
**a** Receiver operator curve (ROC) for the assessment of predictive validity of NRBC measurements. The value that maximized the vertical distance between ROC and diagonal line (representing the highest sum of sensitivity and specificity) was used for calculation of a cutoff value, as described elsewhere [17]. ROC AUC: 0.71; 95% CI 0.66–0.75; *p *< 0.0001. **b** Probability of survival depicted as Kaplan–Meier curves of ARDS patients grouped by NRBC cutoff level of 220 NRBC/µl from ROC analysis. Log-rank test: *p *< 0.001. **c** Receiver operator curves (ROC) for the assessment of predictive validity of NRBCs in comparison to severity of illness scores APACHE, SAPS, SOFA and TISS at ICU admission. NRBC AUC = 0.713 (95% confidence interval = 0.665–0.758); APACHE AUC = 0.626 (95% confidence interval = 0.576–0.675); SAPS AUC = 0.618 (95% confidence interval = 0.567–0.667); SOFA AUC = 0.60 (95% confidence interval = 0.549–0.650); TISS AUC = 0.605 (95% confidence interval = 0.554–0.654). AUC of the NRBC ROC curve was significantly different from those of the other parameters: pairwise comparison: NRBC vs. APACHE: *p* = 0.018; NRBC vs. SOFA: *p* < 0.001; NRBC vs. SAPS: *p* = 0.009; NRBC vs. TISS: *p* = 0.003). *NRBC* nucleated red blood cells; *SAPS* Simplified Acute Physiology Score; *APACHE* Acute Physiology And Chronic Health Evaluation; *SOFA* Sequential Organ Failure Assessment, *TISS* Therapeutic Intervention Scoring System

To further evaluate the predictive quality of NRBCs in ARDS, we calculated a cutoff value according to the Youden’s index [17] (Fig. 4a). A cutoff value of 220 NRBC per µl was found to best distinguish between survival and death (ROC AUC 0.71; 95% CI 0.66–0.75; *p *< 0.0001). Logistic regression analyses with mortality as response identified this cutoff to be an independent risk factor for mortality, revealing a more than tripled risk for ICU death when exceeding this threshold (OR 3.2; 95% CI 1.93–5.35; *p *< 0.0001). With a further increase in NRBC value by 1000/µl, OR increased to 5.8 generating a 5.8-fold increase in the mortality risk (Table 2).

Cumulative survival is presented with Kaplan–Meier curves and revealed a median survival of 85 days in patients with NRBC counts below the respective cutoff level of 220/µl, whereas patients above this level had a median survival time of 29 days (log rank *p *< 0.001; Fig. 4b). A corresponding Cox proportional hazard regression model confirmed these findings. After backward feature selection, a NRBC value above 220/µl was found to be an independent risk factor for mortality, generating a 1.4-fold increase in the risk for ICU death during the observation period (HR 1.41; 95% CI 1.007–1.99; *p *< 0.05). Also, the APACHE II score and pulmonary compliance were identified as independent variables for mortality (Table 2). Median follow-up for all patients was 35 (95% CI 31.75–38.25) days.

Receiver operator curves (ROC) for the assessment of predictive validity of NRBCs in comparison to severity of illness scores APACHE, SAPS, SOFA and TISS at ICU admission are presented in Fig. 4c. Comparing the AUC values, AUC of NRBC was highest (with respect to mortality) and differed significantly from those of the other parameters (pairwise comparison: NRBC vs. APACHE: *p* = 0.018; NRBC vs. SOFA: *p* < 0.001; NRBC vs. SAPS: *p* = 0.009; NRBC vs. TISS: *p* = 0.003) (Fig. 4c).

### Pulmonary gas exchange and severity of organ failure

**Fig. 5:**
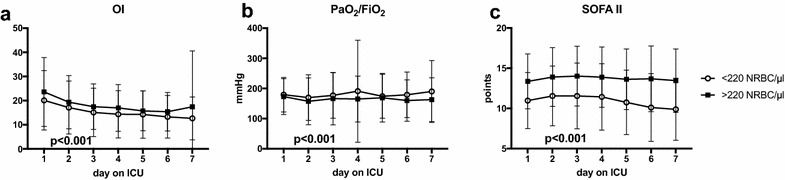
**a** Oxygenation index (OI), **b** PaO_2_/FiO_2_ ratio and **c** SOFA II score in ARDS patients grouped by the NRBC cutoff level of 220 NRBC/µl from ROC analysis in the first consecutive 7 days on the ICU. Open circles = ARDS patients below the threshold of 220 NRBC/µl; filled squares = ARDS patients above the threshold of 220 NRBC/µl; data are presented as mean ± standard deviation; Multivariate nonparametric analysis for longitudinal data, indicated *p* value for comparison between the two groups over time; *n* = 404. *PaO*_*2*_ arterial partial pressure of oxygen; *FiO*_*2*_ inspiratory fraction of oxygen; *OI* oxygenation index, calculated as FiO_2_/PaO_2_ * Pmean; *Pmean* mean airway pressure; *SOFA* Sequential Organ Failure Assessment; *ICU* intensive care unit; *NRBC* nucleated red blood cells

In order to test the discriminatory power of the cutoff value of 220 NRBC/µl regarding ARDS specific parameters, this value was used to group patients for analysis of the typical parameters of gas exchange such as the PaO_2_/FiO_2_ ratio, the oxygenation index (OI) and the severity of organ failure score (SOFA II) on the first consecutive 7 days on the ICU (Fig. 5a–c). Over the course of time, these indices and surrogate parameters of pulmonary gas exchange were constantly more impaired in ARDS patients with NRBC levels above 220/µl compared to ARDS patients with NRBC counts below that threshold. In a multivariate nonparametric longitudinal analysis, all regarded parameters were significantly different between patients above or below the cutoff value of 220 NRBC/µl (*p *< 0.001).

### Risk factors for the occurrence of NRBCs > 220/µl

Finally, we examined independent risk factors for the occurrence of NRBCs above the calculated cutoff value of 220/µl in the peripheral blood of ARDS patients. Multiple logistic regression identified the presence of “severe ARDS” and “pulmonary compliance” to be independent risk factors for the occurrence of NRBCs in the peripheral blood (severe ARDS: OR 1.78, 95% CI 1.14–2.77, *p *= 0.011 and pulmonary compliance: OR 0.98, 95% CI 0.97–0.99, *p *= 0.001). In patients with severe ARDS, the risk of having NRBC values above 220/µl was found to be about 80% higher compared to patients with mild or moderate ARDS. Further, severity of organ failure score (SOFA II) and simplified physiological score (SAPS) at admission were identified as independent risk factors (SOFA: OR = 1.22, 95% CI 1.15–1.30, *p* < 0.001; SAPS: OR = 1.03, 95% CI 1.02–1.04, *p* < 0.001), whereas age, gender, the implementation of extracorporeal gas exchange with ECMO, inspiratory pressures and PaO2/FiO2 ratio did not prove to be independent predictors of NRBC positivity > 220/µl.

## Discussion

This single-center, retrospective cohort study investigated the predictive value of NRBCs on ICU mortality in a broad population of ARDS patients. We found that the presence of NRBCs in the blood was independently associated with a more than doubled risk of ICU death. Moreover, NRBC positivity at ICU admission was an independent predictor of mortality, yielding a more than threefold risk of ICU death. A cutoff level of 220 NRBC/µl was found to be the most accurate value with respect to predictive validity and was associated with a more than tripled risk of ICU death in these ARDS patients. The presence of a severe ARDS alone, i.e., the sole presence of a severely impaired pulmonary gas exchange, was identified as an independent risk factor for NRBC positivity above the threshold of 220/µl.

Our results corroborate previous findings in critically ill patients, suggesting that NRBCs are equally predictive of mortality in ARDS [5, 6, 9, 10, 12–14]. Interestingly, our study demonstrates a much higher prevalence of NRBCs of more than 75% in ARDS patients than in other cohorts. In a recent investigation, de Moura Junior and colleagues found that 54% of patients admitted to a cardiac ICU were NRBC positive [14]; other studies report even lower values of 17.5 and 24.8% in septic or surgical patients [5, 6, 10, 12]. Moreover, peak values of NRBCs found in the present study were much higher than reported elsewhere [5, 6, 10, 12]. The underlying mechanisms that lead to the occurrence of NRBCs in the peripheral blood are not yet completely understood. However, arterial hypoxemia and systemic inflammation with high levels of pro-inflammatory cytokines are suggested as potent triggering factors for the release of NRBCs into the peripheral circulation [8, 9, 18, 19]. Both factors are usually present in critically ill patients with ARDS [20]. We speculate that they affect NRBC levels in a combined and additive manner, which might explain the high prevalence of NRBCs in ARDS patients. In line with this, we found that the sole presence of a “severe ARDS”, which is defined by marked arterial hypoxemia, did significantly increase the risk of NRBC positivity. Further, typical parameters describing the severity of hypoxemia (such as the oxygenation index or the PO_2_/FiO_2_ ratio) were significantly more impaired in patients with high NRBC values. This not only indicates a more severe clinical condition in these ARDS patients, but also links the occurrence of NRBCs in the peripheral blood to arterial hypoxemia.

The population of the present study was characterized by severe medical conditions, as reflected by high APACHE II and SOFA II admission scores. On average, severity scores were higher than in comparable recent studies [14] and even higher than in the ALIVE study, which examined epidemiology and outcomes of ARDS patients across European ICUs [21]. Along that line, the parameters of pulmonary gas exchange and invasiveness of mechanical ventilation indicated severe lung failure in most of our patients. Interestingly, when applying the Berlin definition of ARDS [4], we observed that NRBC positivity was more frequent and NRBC counts were significantly higher in moderate and severe lung failure compared with mild ARDS. Moreover, NRBC-positive patients required longer treatment with mechanical ventilation, had a significantly higher percentage of extracorporeal gas exchange and had a prolonged ICU stay compared with NRBC-negative patients. Pulmonary compliance, which reflects stiffness of the lungs and might be regarded as a surrogate of the extent of the acute lung injury, was significantly lower in NRBC-positive patients. These data support the assumption that the presence of NRBCs in the peripheral blood might be regarded as an accurate marker of severity of illness in ARDS.

Trends in NRBC values over the time course indicated that the disappearance of these cells from the peripheral circulation was protective in comparison with patient subgroups with values that never returned to baseline. This was particularly evident when considering the patients surviving ARDS. Our observations are in line with findings by Shah and colleagues, who described this effect in patients with surgical sepsis [22]. The peak NRBC value tended to occur earlier in patients who survived ARDS, whereas non-surviving patients tended to develop NRBC peak values later during their ICU stay. Although this effect did not reach statistical significance in our study, one may speculate that high NRBC peaks that occur relatively late in the course of the ICU stay might indicate ongoing disease in critically ill patients.

Attempts to predict outcome in critically ill ARDS patients on the basis of clinical parameters play a major role for appropriate treatment decisions, resource allocation and adequate communication. The strategic focus of ARDS research, however, has shifted toward identifying patients at a high risk of developing or dying from ARDS. In this regard, the concept of individualized therapeutic approaches for ARDS patients is promising and might contribute to the improvement in ARDS outcomes [23, 26]. Reliable biomarkers might be both useful for (1) the identification of patients at risk for developing ARDS and (2) for monitoring treatment progress on the ICU. Further prospective clinical studies are needed to determine whether NRBC levels can be used as an ARDS biomarker.

The present study shows that any positive NRBC value was independently associated with a higher risk of ICU death and that increasing NRBC peak values were associated with increasing mortality rates in ARDS patients. This is consistent with numerous previous reports in which detection of NRBCs was associated with a relatively poor prognosis [5–14, 22, 24]. However, in our study some NRBC-positive patients survived and some NRBC-negative patients died, and vice versa. Nevertheless, NRBCs were of high prognostic power in ARDS patients and an independent predictor of mortality, even at ICU admission. Also, our analysis revealed that a cutoff value of 220 NRBC/µl was the most appropriate level to distinguish between survival and death, yielding the highest sensitivity and specificity values. When exceeding this threshold, mortality risk more than tripled in our ARDS population. When considering the high mortality risk being inherent to ARDS, a more than tripled risk of death is of considerable impact [25, 26]. Interestingly, in the present study, NRBCs were not only independent predictors of mortality but also had high discriminatory power with respect to pulmonary gas exchange and organ failure. This supports the view that the presence of NRBCs may serve as an accurate marker of ARDS severity. Moreover, in a recent study by Purtle and colleagues, NRBCs were identified as a robust predictor of postdischarge mortality in critically ill patients who survived hospitalization [27]. Although not specific for ARDS, these data demonstrate that NRBCs might be strongly predictive of outcomes in the critically ill, even after discharge from the ICU. Whether this holds true for ARDS patients needs to be elucidated in prospective studies. Currently, there is no prognostication tool in ARDS involving the NRBC count. It remains unclear whether monitoring of NRBCs and subsequent changes in therapy or treatment based upon that monitoring (e.g., a change of antibiotics or therapeutic procedures), might improve outcomes in ARDS patients. Therefore, prospective randomized controlled trials that incorporate the presence of NRBCs in the blood of ARDS patients may provide important information on how to more effectively use this parameter as a marker of prognosis in ARDS.

The present study has several limitations including its retrospective design and the limited sample size. Also, including only patients with NRBC measurements may have introduced selection bias. In addition, the high prevalence of NRBCs might also be partly attributable to a more frequent monitoring, thereby enabling a higher rate to be detected if NRBCs appear only transiently. Further, the ARDS patients analyzed in our study suffered from severe medical conditions and had very high severity scores. Also, the number of patients with mild ARDS was relatively low. In this regard, mild ARDS might be under-represented to a certain extend. Because different results and/or thresholds might emerge from other patient cohorts, generalization of our results to other patients requires some caution.

## Conclusions

In our cohort of ARDS patients, the presence of NRBCs in the peripheral blood was significantly associated with disease severity and ICU mortality. NRBCs may predict mortality in patients with ARDS. Larger prospective validation studies are required to prove our findings.

## Abbreviations

ARDS: acute respiratory distress syndrome
NRBC: nucleated red blood cells
ICU: intensive care unit
NO: nitric oxide
ECLS: extracorporeal lung support
(vv) ECMO: veno-venous extracoporeal membrane oxygenation
pECLA: pumpless extracorporeal lung assist
APACHE II: Acute Physiology And Chronic Health Evaluation
SAPS: Simplified Acute Physiology Score
SOFA II: Sequential Organ Failure Assessment
TISS-28: Therapeutic Intervention Scoring System-28
Ppeak: positive inspiratory peak pressure
Pmean: mean airway pressure
PEEP: positive end-expiratory pressure
FiO_2_: fraction of inspired oxygen
PaO2: arterial partial pressure of oxygen
ROC: receiver operator curve
AUC: area under the curve
MANOVA: multivariate nonparametric analyses of longitudinal data
